# Leukemia Cutis Exhibiting the Koebner Phenomenon: A Rare Clinical Presentation of Acute Myeloid Leukemia

**DOI:** 10.1155/crdm/6524620

**Published:** 2026-06-26

**Authors:** Megan Hauptman, Joseph S. Durgin, Ahmad Mattour, Shatha Farhan, Mio Nakamura, Michael Goldfarb

**Affiliations:** ^1^ Department of Dermatology, University of Michigan, Ann Arbor, Michigan, USA, umich.edu; ^2^ Department of Hematology, Medical Oncology, Henry Ford, Detroit, Michigan, USA

**Keywords:** acute myeloid leukemia, Koebner phenomenon, leukemia cutis

## Abstract

Leukemia cutis with predilection for sites of prior cutaneous trauma, often described as a Koebner‐like phenomenon, is a rare but recognized occurrence. We present a unique case of an, otherwise, healthy male who developed leukemia cutis at a prior unrelated biopsy site, leading to a new diagnosis of acute myeloid leukemia (AML). Certain AML subtypes with extramedullary propensity, particularly M4 and M5, as seen in our patient, are associated with an increased risk of leukemia cutis. Unlike their medullary counterparts, extramedullary AML cells preferentially migrate to and persist in tissues outside the bone marrow, where the local microenvironment may promote immune escape. Although the localization of leukemia cutis to the biopsy site is consistent with a Koebner‐like response, it remains uncertain whether subclinical leukemic cells were already present at the time of the initial excision; therefore, a definitive causal relationship between trauma and leukemic infiltration cannot be established. Additionally, the prior keratoacanthoma and its excision may have created a localized immunocompromised cutaneous district, or *locus minoris resistentiae*, further predisposing the site to leukemic cell infiltration. The immunologic features of extramedullary AML, including impaired antigen presentation and T‐cell exhaustion, overlap with this concept of localized immune dysregulation and may have facilitated site‐specific leukemic infiltration. New infiltrative plaques at sites of prior skin trauma or biopsy warrant prompt biopsy and histopathological evaluation, even in patients without known hematologic malignancy. Leukemia cutis may represent the first manifestation of underlying acute leukemia, making early recognition critical for timely oncologic referral and treatment.

## 1. Introduction

Leukemia cutis demonstrating tropism for areas of skin trauma (Koebner phenomenon) is a rare but recognized occurrence [1–6]. In leukemia cutis, the Koebner phenomenon is thought to result from trauma‐ or stress‐induced upregulation of chemokine receptors and adhesion molecules at affected sites, which promotes the homing and accumulation of leukemic cells [7, 8]. In all previously reported cases, patients had an established diagnosis of leukemia prior to the development of leukemia cutis at sites of trauma. We present a unique case of an, otherwise, healthy male who developed leukemia cutis at a prior unrelated biopsy site, a presentation that led to a new diagnosis of acute myeloid leukemia (AML). To our knowledge, this represents the first reported case of leukemia cutis arising at a prior biopsy site as the initial manifestation of AML.

## 2. Case Presentation

**FIGURE 1:**
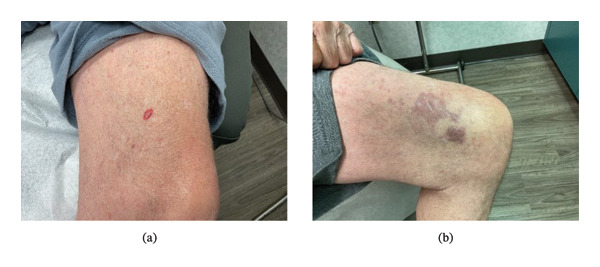
(a) Initial lesion. A 7‐mm erythematous, dome‐shaped keratotic papule on the left inner thigh, later excised and diagnosed as a keratoacanthoma. (b) Leukemia cutis at the prior excision site. Erythematous‐to‐violaceous indurated plaques on the left inner thigh developing 2 months after keratoacanthoma excision.

A 69‐year‐old male with a history of keratinocyte carcinomas presented with a painful, rapidly enlarging lesion on his inner left thigh. He denied systemic symptoms such as fever, chills, and weight change. Physical examination revealed a 7‐mm dome‐shaped, keratotic lesion on the left inner thigh (Figure 1a), with the remainder of his skin examination unremarkable. The lesion was excised in its entirety, and histopathological analysis confirmed keratoacanthoma with clear margins.

**FIGURE 2:**
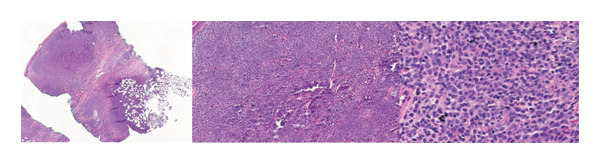
H&E‐stained sections of a biopsy obtained from the area of induration at the prior excision site demonstrate a dense dermal infiltrate of atypical leukemic cells, consistent with leukemia cutis (2X, 10X, 40X).

**FIGURE 3:**
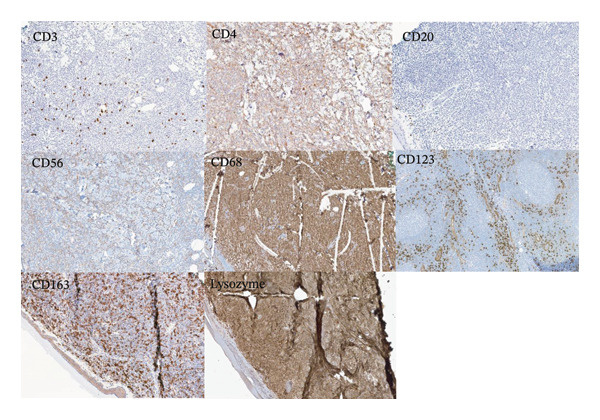
Immunohistochemical (IHC) staining of the inflammatory infiltrate at the site of prior keratoacanthoma excision site. Sections were stained with antibodies against the following markers to characterize the composition of the inflammatory infiltrate: CD3 (T lymphocytes), CD4 (helper T lymphocytes), CD20 (B lymphocytes), CD123 (plasmacytoid dendritic cells), CD163 (M2 macrophages), CD56 (natural killer cells), CD68 (macrophages), and lysozyme (myeloid and monocytic cells).

Two months later, the patient returned with concern for two new skin lesions that had been progressively enlarging over the preceding 2‐3 weeks at the site of the prior keratoacanthoma excision. The patient denied systemic symptoms, including fatigue, night sweats, weight loss, lightheadedness, shortness of breath, and bone, back, or abdominal pain. On examination, two red indurated plaques measuring 5 and 3 cm were observed on the inner left thigh, with the biopsy scar traversing the larger plaque (Figure 1b). A punch biopsy was performed, and histopathology revealed cutaneous involvement by AML with monocytic differentiation, consistent with leukemia cutis (Figures 2 and 3).

**FIGURE 4:**
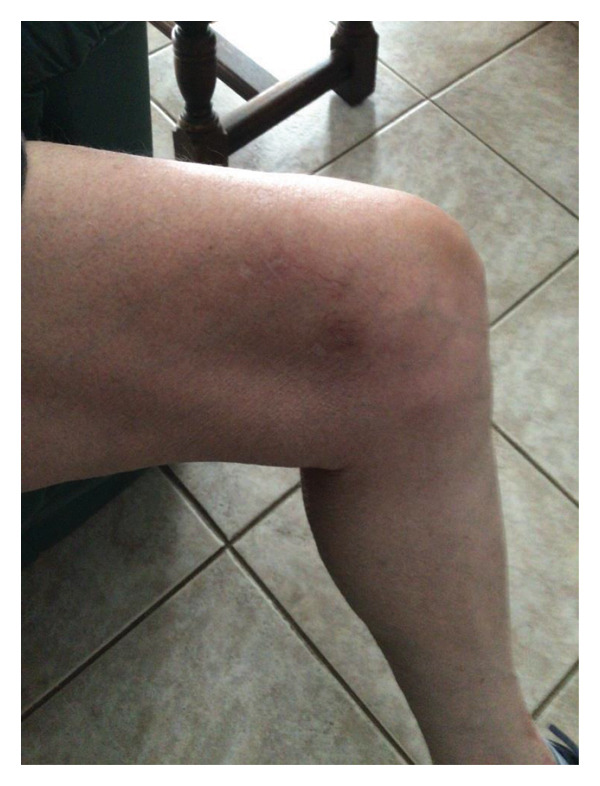
Complete clinical resolution of leukemia cutis at the prior excision site following AML‐directed therapy.

Subsequent staging demonstrated systemic disease. Complete blood count with differential was notable with WBC 31.8 K/μL (17% blasts), RBC 3.91 M/μL, hemoglobin 12.0 g/dL, and hematocrit 35.8%. Peripheral blood showed frequent circulating promonocytes/blast equivalents, absolute and relative monocytosis, normocytic normochromic anemia, and no overt granulocytic dysplasia. Bone marrow aspirate, core biopsy, clot section, and touch imprints confirmed AML with NPM1 mutation and monocytic differentiation. Peripheral blood flow cytometry revealed an expanded monocytic population with heterogeneous CD14 expression comprising approximately 30% of the total events. The patient was admitted for induction chemotherapy and bone marrow transplantation for the treatment of AML. The skin lesions resolved with AML treatment (Figure 4), and post‐transplant bone marrow biopsy demonstrated complete remission with undetectable NPM1 measurable residual disease. As of day 139 following bone marrow transplantation, the patient remained clinically well with no evidence of relapse.

## 3. Discussion

The pathogenesis of extramedullary tropism in AML remains incompletely understood. Emerging evidence suggests that this phenomenon is driven by a combination of altered homing receptor expression on leukemic cells and an enhanced capacity for immune evasion within the extramedullary microenvironment [9]. Certain subtypes of AML, specifically M4 and M5 as seen in our patient, have a significantly increased risk of leukemia cutis [10]. Compared with their medullary counterparts, extramedullary AML cells exhibit increased expression of ICAM1 (CD54) and decreased expression of PECAM1 (CD31), which likely contributes to their preferential migration and retention outside the bone marrow [9].

Moreover, the extramedullary microenvironment promotes immune escape. Transcriptomic analyses have demonstrated the downregulation of HLA Class II molecules, indicative of decreased antigen presentation and evasion of CD4+ T cell surveillance [9]. In addition, T cells associated with extramedullary site tumors display increased markers of exhaustion, including elevated expression of immune checkpoint molecules such as PDCD1 (PD‐1), LAG‐3, and CTLA‐4 [9]. Collectively, these findings suggest that extramedullary AML persistence and proliferation are supported by a specialized microenvironment characterized by impaired antigen presentation and T cell exhaustion, thereby facilitating immune escape.

The localization of leukemia cutis to the prior excisional biopsy site in our patient is consistent with a Koebner‐like response, potentially mediated by trauma‐induced chemokine and adhesion molecule upregulation. However, classic koebnerization implies lesion development in patients with an established underlying disease and malignancy‐associated cases are often classified as questionable or pseudo‐koebnerization [11, 12]. Because the biopsy preceded the AML diagnosis, subclinical or circulating leukemic cells may already have been present. Thus, trauma may have facilitated homing of pre‐existing leukemic cells rather than caused de novo infiltration.

An alternative, complementary framework is Ruocco’s concept of the immunocompromised cutaneous district (ICD), or *locus minoris resistentiae* [13]. An ICD is a site of localized immune dysregulation after prior injury that predisposes to infections, tumors, or dysimmune reactions. Mechanisms include impaired lymphatic drainage, altered immune cell trafficking, scar‐related reduction in lymphatic regeneration, and neuroimmune dysregulation [14, 15]. In our patient, the antecedent keratoacanthoma, excisional biopsy, and resultant scar may have created a localized ICD, providing a permissive niche for leukemic cell infiltration. This concept complements the Koebner‐like mechanism by suggesting that the prior biopsy site represented a region of impaired local immune surveillance, creating a permissive microenvironment for circulating leukemic cells to engraft and proliferate.

Importantly, the immunologic alterations of AML overlap with *locus minoris resistentiae* and may have contributed to site‐specific infiltration. Extramedullary AML is associated with HLA Class II downregulation and T‐cell exhaustion, paralleling the diminished immune surveillance of an ICD [9, 13]. Thus, the excisional biopsy site may have provided a trauma‐conditioned niche with lymphatic, neuroimmune, and scar‐associated immune impairment that facilitated leukemic engraftment. Infiltrating AML cells may then have further amplified local immunosuppression, promoting persistence and proliferation.

Leukemia cutis is associated with a poor prognosis, with several series reporting a median survival of 5–7 months after diagnosis [16]. In AML, leukemia cutis has been associated with approximately a twofold increase in the odds of leukemia‐related death and reduced 5‐year survival compared with AML patients without leukemia cutis [10]. Accordingly, clinicians should maintain a high index of suspicion and a low threshold for biopsy when new infiltrative plaques develop at the sites of prior cutaneous trauma or biopsy, including in patients without a known hematologic malignancy. Prompt histopathological evaluation is essential, as early identification of leukemia cutis not only confirms the diagnosis but may also serve as the initial indicator of underlying acute leukemia. Early recognition facilitates timely referral to hematology/oncology and initiation of appropriate therapy, thereby improving patient outcomes.

## Funding

The authors have nothing to report.

## Disclosure

The authors have nothing to report.

## Consent

The authors obtained written patient consent for use of their photographs and medical information to be published online with the understanding that this information may be publicly available and discoverable via search engines.

## Conflicts of Interest

The authors declare no conflicts of interest.

## Data Availability

The data that support the findings of this study are available from the corresponding author upon reasonable request.
